# Food and nutrition literacy and diet self-efficacy among patients with liver cancer: latent profile analysis, associated factors, and an exploratory examination of gender differences

**DOI:** 10.3389/fnut.2026.1839963

**Published:** 2026-06-24

**Authors:** Ziang Liu, Tianyu She, Shaojian Mo, Wanni Wei, Yuting Guo, Yunfeng Liu, Yi Zhang

**Affiliations:** 1 Department of Endocrinology, First Hospital of Shanxi Medical University, Shanxi Medical University, Taiyuan, China; 2 Department of Pharmacology, Shanxi Medical University, Taiyuan, Shanxi, China; 3 Biliary and Pancreatic Surgery Department, Shanxi Bethune Hospital, Shanxi Academy of Medical Sciences, Third Hospital of Shanxi Medical University, Tongji Shanxi Hospital, Taiyuan, Shanxi, China; 4 Department of Medical Imaging Function, Xi'an Electric Power Central Hospital, Xi'an, Shaanxi, China; 5 Guangzhou Medical University, Guangzhou, China; 6 Shanxi Province Cancer Hospital/Shanxi Hospital Affiliated to Cancer Hospital, Chinese Academy of Medical Sciences/Cancer Hospital Affiliated to Shanxi Medical University, Taiyuan, Shanxi, China

**Keywords:** cancer-related fatigue, diet self-efficacy, food and nutrition literacy, gender differences, patients with liver cancer, social support

## Abstract

Patients with liver cancer frequently experience a heightened risk of malnutrition and a substantial symptom burden attributable to antitumor treatment. Food and nutrition literacy and diet self-efficacy represent critical psychobehavioral competencies that underpin dietary decision-making and the sustained adoption of healthy eating practices in this population. Nevertheless, few studies have delineated the latent profile typologies of these two constructs or their associated factors, and preliminary gender-stratified explorations remain conspicuously absent. To address these gaps, the present study employed a cross-sectional survey design, recruiting a convenience sample of 456 patients with liver cancer from three tertiary-level hospitals between April and October 2025. Data were collected using the Food and Nutrition Literacy Questionnaire, the Diet Self-Efficacy Scale, the Cancer Fatigue Scale, and the Social Support Scale. Results indicated that the optimal model for the full sample yielded a two-profile solution: a “Low Food and Nutrition Literacy–Low Diet Self-Efficacy” profile (47.37%) and a “High Food and Nutrition Literacy–High Diet Self-Efficacy” profile (52.63%). As an exploratory supplementary analysis, gender-stratified latent profile analyses were conducted; however, the substantial reduction in subgroup sample sizes following stratification by sex precluded the identification of statistically robust optimal profile solutions for either male or female subsamples. Binary logistic regression revealed that cancer-related fatigue constituted an independent risk factor for membership in the low-level profile, whereas social support served as an independent protective factor. Educational attainment, place of residence, and monthly personal income also exhibited partially significant predictive effects. Accordingly, clinical nutrition interventions should prioritize fatigue management and the reinforcement of social support, and stratified, precision-targeted interventions should be implemented on the basis of patients’ nutritional behavioral competency profiles.

## 1 Introduction

Liver cancer ranks among the most prevalent and lethal gastrointestinal malignancies worldwide (1, 2). According to the Global Cancer Statistics 2022 report, approximately 865,000 new cases of liver cancer and 758,000 liver cancer–related deaths were recorded globally, with China alone accounting for over 50% of incident cases (3, 4). Previous research has established that the etiology of liver cancer is closely linked to chronic hepatitis B virus infection, hepatitis C virus infection, alcoholic liver disease, and non-alcoholic fatty liver disease, among other factors (5–7), and the majority of patients present with intermediate- or advanced-stage disease at the time of diagnosis (8). Consequently, the overall prognosis of patients with liver cancer remains poor, with low five-year survival rates (9). Of particular importance, the liver serves as the central organ of systemic metabolism, subserving critical physiological functions including protein synthesis, lipid metabolism, glycogen storage, and the activation of multiple vitamins (10). Hepatic dysfunction therefore directly impairs the digestion, absorption, and utilization of nutrients, thereby precipitating serious complications such as malnutrition, sarcopenia, and cachexia (11, 12). Evidence indicates that approximately 50 to 80% of patients with liver cancer develop varying degrees of malnutrition during the disease trajectory, and deterioration in nutritional status not only compromises tolerance to antitumor therapeutic regimens but also elevates the risk of postoperative complications, attenuates immune function, and adversely affects overall survival and quality of life (13–15). How to effectively improve the nutritional status of patients with liver cancer and facilitate the establishment of scientifically sound dietary behaviors in daily life has thus emerged as a pressing issue in oncology nursing.

Food and nutrition literacy denotes the comprehensive capacity of an individual to access, comprehend, appraise, and apply food- and nutrition-related information in order to make informed and healthful dietary decisions (16, 17). Its core dimensions encompass nutritional knowledge, food label interpretation skills, meal planning competence, and the critical evaluation of food and nutrition information (18, 19). Among oncology populations, higher levels of food and nutrition literacy have been demonstrated to be significantly associated with superior dietary quality, improved nutritional status, and a reduced risk of tumor-related complications, serving as the cognitive prerequisite and informational foundation that drives patients’ proactive adoption of evidence-based dietary behaviors (20). Diet self-efficacy reflects an individual’s perceived confidence and competence in adhering to a healthy eating plan when confronted with diverse and challenging dietary situations (21, 22). In essence, it constitutes a context-specific, behavior-specific form of self-efficacy that exerts an independent and central predictive function in dietary behavior change through the regulation of cognitive–psychological processes including motivational activation, goal setting, and behavioral maintenance. Prior research has confirmed that elevated levels of diet self-efficacy significantly enhance cancer patients’ capacity to overcome treatment-related dietary barriers such as appetite loss, nausea, and dysphagia, and serve as the psychological driving force for improving adherence to medical nutrition therapy and sustaining healthy dietary patterns (23, 24). In the specific context of liver cancer, the convergence of metabolic derangements and malabsorption secondary to hepatic dysfunction, systemic inflammation–mediated appetite suppression (25, 26), and the complex adverse effects of multiline antitumor therapies collectively poses multifaceted challenges to patients’ food and nutrition literacy and diet self-efficacy. More importantly, a mutually reinforcing synergistic relationship may exist between these two constructs. Specifically, higher food and nutrition literacy furnishes patients with a robust cognitive foundation of scientific nutritional knowledge, which in turn facilitates enhanced self-efficacy perceptions in complex dietary situations, whereas stronger diet self-efficacy further promotes the active translation of nutritional knowledge into sustained dietary behavior change, ultimately engendering a positive behavioral feedback loop. To date, however, research that systematically evaluates the joint distributional characteristics and within-group heterogeneity of food and nutrition literacy and diet self-efficacy in patients with liver cancer remains notably scarce, a gap that constrains a thorough understanding of the overall landscape of nutrition-related behaviors in this population.

Cancer-related fatigue is defined as a persistent, function-disrupting subjective experience of exhaustion related to cancer itself or its treatment, characterized by an inability to be alleviated by adequate rest (27, 28). Among patients with liver cancer, cancer fatigue is reported in 60 to 90% of cases (29, 30), driven by tumor-induced systemic inflammatory responses, metabolic abnormalities resulting from hepatic impairment, and the cumulative effects of multiline antitumor therapies. Cancer-related fatigue can exert a profound negative influence on both food and nutrition literacy and diet self-efficacy (31, 32). On the one hand, persistent fatigue depletes patients’ limited cognitive resources and attentional reserves, undermining their capacity to proactively seek, comprehend, and critically evaluate nutritional information, thereby diminishing food and nutrition literacy (33, 34). On the other hand, the physical exhaustion and psychological frustration accompanying fatigue markedly erode patients’ willpower and confidence to plan and consistently implement healthy dietary regimens, producing a pronounced inhibitory effect on diet self-efficacy (35). Social support, as another pivotal psychosocial resource variable, plays an indispensable facilitative role in the psychological coping and health behavior maintenance of oncology patients (36, 37). Adequate social support can furnish patients with liver cancer with external channels for acquiring nutrition-related knowledge and skills (37), bolster their beliefs in overcoming dietary barriers through the reinforcement of positive feedback mechanisms for behavior change, and effectively attenuate fatigue perception and emotional stress responses, thereby enhancing food and nutrition literacy at the cognitive level and strengthening diet self-efficacy at the motivational level. Although existing research has preliminarily documented a positive association between social support and self-management behaviors in general cancer populations (38, 39), a systematic and in-depth empirical investigation of the differential mechanisms through which cancer-related fatigue and social support influence food and nutrition literacy and diet self-efficacy in patients with liver cancer is currently lacking.

Gender constitutes an important sociobiological variable that shapes the biological characteristics, clinical outcomes, and health-related behaviors of oncology patients (40, 41). Prior research has established that the incidence of liver cancer in males is approximately two to three times that in females (42, 43), a disparity potentially attributable to systematic differences across multiple dimensions, including androgen levels versus the protective effects of estrogen, hepatitis B virus carrier rates and patterns of chronic hepatitis progression (44, 45), risk exposure behaviors such as alcohol consumption and smoking, as well as health cognitive orientations, nutritional behavioral habits, and patterns of healthcare resource utilization shaped through gender socialization processes (46, 47). Female patients generally demonstrate advantages over males in nutritional knowledge, proactive health information seeking, and dietary self-management awareness (48); however, they are also more susceptible to interference from emotional eating tendencies, caregiving role burden, and societal gender role expectations (49). Treating the liver cancer patient population as homogeneous while disregarding gender differences would obscure the inherent heterogeneity in the joint dimensions of food and nutrition literacy and diet self-efficacy across sexes, thereby attenuating the precision and real-world effectiveness of intervention designs. Accordingly, the present study adopts latent profile analysis—a person-centered finite mixture modeling approach—that identifies latent heterogeneous subgroups within the study sample through a probabilistic classification mechanism based on continuous measurement variables, thereby revealing the inherent typological structure of individuals along multivariable joint dimensions (50).

Although previous research has generated a degree of evidence from various perspectives on the nutritional behaviors, diet self-efficacy, and associated factors in oncology patients, several critical research gaps remain to be addressed. First, prior studies have predominantly examined food and nutrition literacy and diet self-efficacy as mutually independent, unidimensional variables, failing to adopt an integrative perspective to reveal the latent typological structure of their co-variation and the subgroup distribution patterns within a given patient population, thereby inadequately capturing the inherent heterogeneity of patients’ nutrition-related behaviors. Second, the systematic application of a gender-differential perspective in research on food and nutrition literacy and diet self-efficacy among oncology patients has been extremely limited, and gender-stratified latent profile analyses of subgroup heterogeneity remain entirely uncharted. Third, the joint influence mechanisms of cancer-related fatigue and social support on food and nutrition literacy and diet self-efficacy, as well as their differential pathways across male and female patients with liver cancer, have yet to be subjected to systematic empirical testing.

To address these research gaps, the present study targeted hospitalized and outpatient patients with liver cancer as the study population and employed food and nutrition literacy and diet self-efficacy as the core latent classification variables. Latent profile analysis was used to identify the latent subgroup typologies and distributional characteristics of nutrition-related behaviors among patients with liver cancer. Building on this foundation, the study systematically investigated the differential influences of cancer-related fatigue, social support, and multidimensional sociodemographic and clinical characteristics on latent profile membership. As an exploratory supplementary objective, latent profile analyses were independently conducted within the male and female subsamples to provide a preliminary examination of whether the latent typological structures differ between the sexes under a gender-stratified framework. The anticipated findings of this study are expected to deepen the theoretical understanding of the inherent heterogeneity governing nutrition-related behaviors in patients with liver cancer and to provide a robust evidence base for clinical nutritionists and oncology healthcare professionals to design precision-targeted, subgroup-specific nutrition intervention programs that are sensitive to gender differences, thereby advancing the systematization and individualization of nutritional management practices for patients with liver cancer.

## 2 Method

### 2.1 Study design

This study employed a cross-sectional survey design to identify latent subgroup profiles of food and nutrition literacy and diet self-efficacy among patients with liver cancer through LPA, and to systematically examine the differential effects of cancer-related fatigue, social support, and multidimensional sociodemographic and clinical characteristics on latent profile membership. Additionally, exploratory supplementary analyses were conducted by performing LPA independently within male and female subgroups to preliminarily investigate sex-based differences in the latent profile structure. This study was reviewed and approved by the Medical Ethics Committee of Shanxi Bethune Hospital (No.: SBQLL-2025-253), and written informed consent was obtained from all participants.

### 2.2 Participant recruitment

A convenience sampling approach was adopted to recruit participants between April and October 2025 from the oncology inpatient wards and outpatient clinics of three tertiary (Grade A) hospitals in Shanxi Province, China. Data were collected using a combination of face-to-face structured interviews and self-administered paper-based questionnaires. Patients with adequate visual acuity and a sufficient level of education who were capable of independent reading completed the questionnaires on their own. For older patients or those with impaired vision or limited literacy, research staff read each questionnaire item aloud and faithfully recorded the patients’ responses. A research team member was present throughout the entire data collection process to supervise and assist, thereby ensuring accurate comprehension of each item. Prior to data collection, all participants were fully informed of the study objectives, data confidentiality protocols, and their right to withdraw at any time, and they voluntarily provided written informed consent.

### 2.3 Inclusion and exclusion criteria

Inclusion criteria: (1) histopathologically confirmed diagnosis of primary hepatocellular carcinoma; (2) age ≥ 18 years; (3) clear consciousness and normal mental status, with basic Chinese reading comprehension ability sufficient to complete the questionnaire independently or with assistance; and (4) voluntary participation in the study with provision of written informed consent.

Exclusion criteria: (1) presence of a concurrent primary malignancy or a history of treatment for another malignant neoplasm; (2) clinically diagnosed severe cognitive impairment or severe psychiatric disorder that would compromise the validity of questionnaire responses; (3) critically ill status, an episode of severe acute complications, or an estimated life expectancy of less than three months; (4) severe hearing impairment, aphasia, or other communication barriers precluding completion of the interview; and (5) response patterns exhibiting pronounced regularity or a total response time of less than two minutes.

### 2.4 Study sample

A total of 489 participants were initially recruited. Of these, 33 were excluded for failing to meet the eligibility criteria: 13 had a concurrent primary malignancy, 9 had an estimated life expectancy of less than three months, and 11 exhibited excessively short response times or patterned responding. Consequently, the final analytic sample comprised 456 participants, yielding an effective response rate of 93.25%. Among them, 340 (74.6%) were male and 116 (25.4%) were female. A total of 311 patients (68.2%) were married, and the largest proportion of patients had attained a junior high school or senior high school education (*n* = 184, 40.4%). Detailed demographic characteristics are presented in Table 1.

**Table 1:** Summary of patient demographic characteristics.

Variables	Items	Number	Proportion (%)
Gender	Male	340	74.6
	Female	116	25.4
Marital status	Married	311	68.2
	Unmarried	45	9.9
	Divorced	56	12.3
	Widowed	44	9.6
Educational background	Primary school and below	139	30.5
	Junior high school - Senior high school	184	40.3
	Bachelor’s degree and above	133	29.2
Employment status	Retirement	109	23.9
	Unemployed	161	35.3
	Employed	186	40.8
Place of residence	Urban	291	63.8
	Rural	165	36.2
Monthly personal income	≤4,000 RMB	200	43.8
	4,001–8,000 RMB	159	34.9
	≥ 8,001 RMB	97	21.3
Liver cancer stage	Stage I	60	13.2
	Stage II	129	28.3
	Stage III	189	41.4
	Stage IV	78	17.1
Child–Pugh classification of liver function	A classification	221	48.5
	B classification	167	36.6
	C classification	68	14.9
Cirrhosis status	Yes.	291	63.8
	No.	165	36.2
Time since diagnosis	≤6 month	144	31.6
	6–12 month	112	24.6
	≥1 years	200	43.9
Line of antitumor therapy	Not receiving treatment	44	9.7
	One line	169	37.1
	Two line	142	31.1
	Three line and above	101	22.1
Smoking behavior	Yes.	251	55.0
	No.	205	45.0
Alcohol behavior	Yes.	204	44.7
	No.	252	55.3
Age	M ± SD	56.85 ± 8.707	

### 2.5 Instruments tools

#### 2.5.1 Food and nutrition literacy questionnaire for Chinese adults (FNLQ-CA)

Food and nutrition literacy was assessed using the Food and Nutrition Literacy Questionnaire for Chinese Adults (FNLQ-CA), developed by Zhang et al. (51) and systematically validated for reliability and validity in Chinese adult populations. The questionnaire consists of 20 core items spanning four dimensions: food and nutrition knowledge and comprehension, food acquisition and selection skills, food preparation and processing skills, and healthy eating behaviors. Items are rated on a five-point Likert scale (1 = strongly disagree, 5 = strongly agree); a sample item is “A good dietary pattern is the foundation of adequate nutrition.” Total scores range from 20 to 100, with higher scores indicating a higher level of food and nutrition literacy. In the present study, the Cronbach’s *α* coefficient for the FNLQ-CA was 0.931.

#### 2.5.2 Diet self-efficacy scale

Diet self-efficacy was measured using a Chinese adaptation of the Diet Self-Efficacy Scale originally developed by Hickey et al. (52). The Chinese version, translated and psychometrically validated by Chen and Shao (53) following a five-step back-translation protocol, was employed. The scale systematically assesses an individual’s perceived confidence in adhering to a healthy dietary regimen across a variety of real-life eating situations; a sample item is “Maintaining a healthy diet when no one else is at home.” The instrument comprises 16 unidimensional items scored on a five-point scale (1 = very low confidence, 5 = very high confidence). Total scores range from 16 to 80, with higher scores reflecting greater diet self-efficacy. In the present study, the Cronbach’s *α* coefficient for the Diet Self-Efficacy Scale was 0.909.

#### 2.5.3 Cancer-related fatigue scale

Cancer-related fatigue was measured using the Cancer Fatigue Scale (CFS) developed by Okuyama et al. (54). The Chinese version, translated and validated by Zhang et al. (55), was adopted. The scale comprises 15 items assessing three dimensions: physical fatigue, affective fatigue, and cognitive fatigue. A sample item is “Do you feel so fatigued that you do not know what to do?” Each item is rated on a five-point scale (1 = not at all, 5 = very much). Higher dimension and total scores indicate more severe fatigue symptoms in the corresponding domain. In the present study, the Cronbach’s *α* coefficient for the CFS was 0.898.

#### 2.5.4 Multidimensional scale of perceived social support (MSPSS)

Social support was assessed using the Multidimensional Scale of Perceived Social Support (MSPSS) developed by Zimet et al. (56). The Chinese version, translated by Huang et al. (57), has been validated for cultural appropriateness and reliability in Chinese cancer populations. The MSPSS evaluates subjectively perceived support from distinct relational sources and consists of 12 items organized into three dimensions: support from significant others, support from family, and support from friends. Each item is scored on a five-point Likert scale (1 = strongly disagree, 5 = strongly agree). Total scores range from 12 to 60, with higher scores indicating a greater level of perceived social support from the corresponding source. In the present study, the Cronbach’s *α* coefficient for the MSPSS was 0.889.

### 2.6 Statistical analysis

Data analyses were performed using SPSS version 27.0 and Mplus version 8.3. First, preliminary analyses were conducted, including reliability analysis, common method bias testing, descriptive statistics, and correlation analysis. Subsequently, the individual items of the food and nutrition literacy and diet self-efficacy measures were entered as indicator variables for latent classification. Model parameters were estimated using the expectation–maximization (EM) algorithm with the robust maximum likelihood estimator (MLR), which is resilient to non-normality in the data. A series of competing models specifying one through five latent profiles were fitted sequentially to determine the optimal latent profile solution for the full sample. Concurrently, the complete LPA procedure was independently replicated in the male and female subsamples within a sex-comparative framework to systematically elucidate sex-specific heterogeneity in the latent subgroup structure of food and nutrition literacy and diet self-efficacy among patients with liver cancer. Fourth, once the optimal number of latent profiles was determined, latent profile membership served as the outcome variable, and chi-square tests or independent-samples t-tests were employed to examine differences in sociodemographic characteristics, clinical features, and psychosocial factors across the identified profiles. Fifth, binary logistic regression analysis was conducted to evaluate the independent predictive effects of sociodemographic characteristics, clinical features, and key psychosocial factors on latent profile membership, with odds ratios and their corresponding 95% confidence intervals (95% CIs) reported for each variable.

## 3 Results

### 3.1 Common method bias testing

Common method bias was systematically evaluated using Harman’s single-factor test. The unrotated principal component analysis revealed that the first extracted common factor accounted for 22.368% of the total variance, which was substantially below the 40% critical threshold, indicating that the data in the present study were not subject to serious common method bias.

### 3.2 Descriptive statistics and correlation analysis

Descriptive statistics and bivariate correlations among the core study variables are presented in Table 2. Skewness values for the core study variables ranged from −0.143 to 0.019, and kurtosis values ranged from −0.238 to 0.013, suggesting that all study variables approximated a normal distribution. Correlation analysis revealed a moderate and statistically significant positive association between food and nutrition literacy and diet self-efficacy (r = 0.393, *p* < 0.001), indicating an intrinsic synergy between enhanced nutritional cognitive capacity and greater confidence in executing dietary behaviors. Food and nutrition literacy was significantly and negatively correlated with cancer-related fatigue (r = −0.307, *p* < 0.001) and significantly and positively correlated with social support (r = 0.382, *p* < 0.001). Similarly, diet self-efficacy was significantly and negatively correlated with cancer-related fatigue (r = −0.283, *p* < 0.001) and significantly and positively correlated with social support (r = 0.340, *p* < 0.001). In addition, cancer-related fatigue and social support were significantly and negatively correlated with each other (r = −0.350, *p* < 0.001).

**Table 2:** Descriptive statistics and bivariate correlations among core study variables.

Variables	M	SD	Skewness	Kurtosis	1	2	3	4
1. Food and nutrition literacy	3.219	0.590	−0.143	−0.104	1			
2. Diet self-efficacy	3.003	0.593	0.019	0.013	0.393***	1		
3. Cancer-related fatigue	3.512	0.537	−0.036	−0.238	−0.307***	−0.283***	1	
4. Social support	3.272	0.592	−0.053	−0.208	0.382***	0.340***	−0.350***	1

***p < 0.001; M = Mean; SD = Standard deviation.

### 3.3 Latent profile analysis of food and nutrition literacy and diet self-efficacy

Individual items from the food and nutrition literacy questionnaire and the diet self-efficacy scale served as indicator variables for latent classification. Using the expectation–maximization algorithm with the robust maximum likelihood estimator, a series of competing models specifying one through five latent profiles were fitted sequentially. Model fit indices for each solution are summarized in Table 3.

**Table 3:** Model fit indices for latent profile analysis of food and nutrition literacy and diet self-efficacy.

Indices	Profile 1	Profile 2	Profile 3	Profile 4	Profile 5
AIC	43284.709	**40569.987**	39590.053	38811.013	38515.784
BIC	43581.528	**41019.339**	40191.937	39565.429	39422.732
aBIC	43353.023	**40673.408**	39728.58	38984.646	38724.523
Entropy	**—**	**0.909**	0.921	0.915	0.911
LMR (p)	**—**	**0.004**	0.305	0.261	0.201
BLRT (p)	**—**	**< 0.001**	< 0.001	< 0.001	< 0.001
Class 1	**—**	**47.37%**	19.52%	15.57%	11.18%
Class 2	**—**	**52.63%**	57.89%	34.65%	22.59%
Class 3	**—**	**—**	22.59%	27.41%	25.00%
Class 4	**—**	**—**	**—**	22.37%	25.22%
Class 5	**—**	**—**	**—**	**—**	16.01%

AIC = Akaike information criterion; BIC = Bayesian information criterion; aBIC = adjusted BIC; LMR = Lo–Mendell–Rubin test; BLRT = Bootstrapped likelihood ratio test. Bold text indicates the optimal latent profile.

With respect to information criteria, as the number of profiles increased from one to five, the AIC decreased continuously from 43,284.709 to 38,515.784, the BIC decreased from 43,581.528 to 39,422.732, and the aBIC decreased from 43,353.023 to 38,724.523. This consistently declining trend reflected progressive improvement in the model’s capacity to describe the data. Regarding classification precision, entropy values for the two- through five-profile solutions ranged from 0.909 to 0.921, all exceeding the 0.90 threshold indicative of excellent classification accuracy, thereby confirming that each multi-profile model assigned individuals to profiles with high confidence. The Lo–Mendell–Rubin likelihood ratio test (LMR-LRT) indicated that the two-profile model represented a statistically significant improvement over the one-profile baseline model (*p* = 0.004); however, the LMR-LRT comparing the three-profile model with the two-profile model did not reach statistical significance (*p* = 0.305), suggesting that the addition of a third profile did not yield a substantive improvement in model fit beyond the two-profile solution. The bootstrap likelihood ratio test (BLRT) was highly significant (*p* < 0.001) for all two- through five-profile models, further confirming the superiority of multi-profile solutions over the single-profile model. Based on these findings, the two-profile model was determined to be the optimal latent profile solution for the current sample.

**Figure 1:**
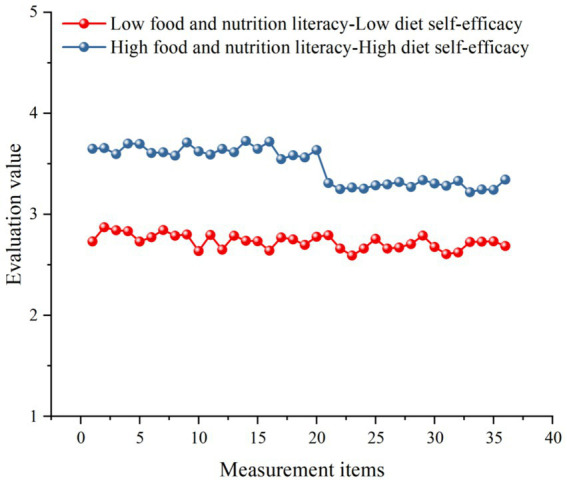
Profile plot of the optimal latent profile solution for food and nutrition literacy and diet self-efficacy.

Based on the foregoing results, the optimal profile solution for food and nutrition literacy and diet self-efficacy was visualized using Origin 2021 software, as shown in Figure 1.

Profile 1: “Low food and nutrition literacy–low diet self-efficacy” (*n* = 216, 47.37%). This subgroup exhibited lower scores on items assessing food and nutrition knowledge and comprehension, food acquisition and selection skills, food preparation and processing skills, and healthy eating behaviors. Moreover, when confronted with a range of real-life dietary barrier scenarios—such as time pressure, eating out, holiday food temptations, and family members not adhering to a healthy diet—these individuals reported markedly insufficient subjective confidence in maintaining a healthy dietary regimen, demonstrating a dual disadvantage characterized by weak nutritional cognition and diminished confidence in dietary behavior execution.

Profile 2: “High food and nutrition literacy–high diet self-efficacy” (*n* = 240, 52.63%). This subgroup attained relatively higher scores across all food and nutrition literacy items and on self-efficacy items pertaining to various dietary barrier scenarios. These individuals possessed a more systematic reservoir of nutritional knowledge and stronger confidence in executing healthy eating behaviors, exhibiting a pattern in which nutritional cognitive capacity and behavioral motivational resources were concurrently advantageous.

### 3.4 Exploratory supplementary analysis: preliminary gender-stratified latent profile examination

As an exploratory supplementary analysis, latent profile analysis was independently conducted within the male (*n* = 340) and female (*n* = 116) subsamples, with one- through five-profile models estimated for each subgroup. Results are summarized in Tables 4, 5, respectively. It should be explicitly noted prior to presenting these results that the sample sizes of the sex-stratified subgroups were substantially reduced relative to the full sample, which may have imposed substantive limitations on the statistical power of the LMR-LRT, thereby making it difficult to detect significant improvements in fit when comparing adjacent models. The following findings should therefore be interpreted as exploratory and preliminary and should not be treated as definitive conclusions regarding sex-based differences in latent profile structure; they await corroboration in future studies with adequately powered sex-stratified samples.

**Table 4:** Model fit indices for latent profile analysis of food and nutrition literacy and diet self-efficacy (male subsample).

Indices	Profile 1	Profile 2	Profile 3	Profile 4	Profile 5
AIC	32434.436	30282.261	29484.798	28854.840	28584.461
BIC	32710.120	30699.616	30043.824	29555.537	29426.829
aBIC	32481.723	30353.847	29580.684	28975.026	28728.948
Entropy	**—**	0.909	0.935	0.927	0.938
LMR (p)	**—**	0.270	0.390	0.165	0.129
BLRT (p)	**—**	< 0.001	< 0.001	< 0.001	< 0.001
Class 1	**—**	51.47%	17.70%	15.00%	12.65%
Class 2	**—**	48.53%	58.89%	30.29%	27.06%
Class 3	**—**	**—**	23.41%	31.77%	27.06%
Class 4	**—**	**—**	**—**	22.94%	17.94%
Class 5	**—**	**—**	**—**	**—**	15.29%

**Table 5:** Model fit indices for latent profile analysis of food and nutrition literacy and diet self-efficacy (female subsample).

Indices	Profile 1	Profile 2	Profile 3	Profile 4	Profile 5
AIC	10929.420	10407.331	10211.994	10068.804	10023.637
BIC	11127.678	10707.473	10614.018	10572.711	10629.427
aBIC	10900.089	10362.928	10152.518	9994.256	9934.016
Entropy	**—**	0.924	0.928	0.955	0.958
LMR (p)	**—**	0.149	0.368	0.195	0.823
BLRT (p)	**—**	< 0.001	< 0.001	< 0.001	< 0.001
Class 1	**—**	45.69%	14.66%	16.38%	20.69%
Class 2	**—**	54.31%	46.55%	28.45%	14.66%
Class 3	**—**	**—**	38.79%	29.45%	33.62%
Class 4	**—**	**—**	**—**	26.72%	25.86%
Class 5	**—**	**—**	**—**	**—**	5.17%

As shown in Table 4, in the male subsample, the AIC, BIC, and aBIC decreased continuously as the number of profiles increased from one to five, suggesting that additional profiles improved model fit. However, the LMR-LRT failed to reach statistical significance for any of the two- through five-profile models (all *p* > 0.05), providing no clear statistical evidence that the addition of a profile significantly improved upon the preceding model. Meanwhile, the BLRT was significant across all two- through five-profile models, indicating that more complex models were statistically superior to more parsimonious models at the likelihood level; however, this test is known to be sensitive to sample size and model complexity in practice and may remain persistently significant as the number of profiles increases. Entropy values in the male subsample remained at high levels across the two- through five-profile solutions, indicating that all profile solutions achieved relatively clear individual classification. Thus, the male subsample exhibited a pattern characterized by continuously improving information criteria, persistently significant BLRT, non-significant LMR-LRT, and high entropy, suggesting that model fit improvement did not reach a clear inflection point at the statistical level, making it difficult to determine a single optimal number of profiles based on any individual index.

As shown in Table 5, in the female subsample, the AIC, BIC, and aBIC likewise decreased continuously as the number of profiles increased from one to five, indicating progressive improvement in fit with higher-order profile solutions. Consistent with the male subsample, the LMR-LRT did not reach statistical significance for any of the two- through five-profile models (all *p* > 0.05), failing to support the conclusion that the stepwise addition of profiles yielded significant improvement. The BLRT was significant across all two- through five-profile models (all *p* < 0.001), suggesting that more complex models were statistically superior to more parsimonious models at the likelihood level, although the same issue of persistent significance and limited ability to pinpoint the optimal number of profiles was present. Entropy values in the female subsample ranged from 0.924 to 0.958 across the two- through five-profile models, indicating overall excellent classification accuracy. Therefore, the female subsample also displayed a pattern of continuously declining information criteria, persistently significant BLRT, non-significant LMR-LRT, and high entropy, leaving the profile number selection without singular, unambiguous, and consistent statistical support.

The LPA results for both gender-stratified subsamples exhibited a consistent statistical pattern of continuously declining information criteria, persistently significant BLRT, and consistently non-significant LMR-LRT, precluding the unambiguous identification of an optimal number of profiles via commonly employed statistical criteria. Considered within the methodological context, this outcome largely reflects the objective constraint that reduced subsample sizes following sex stratification imposed on the statistical power of the LMR-LRT, rather than necessarily implying the absence of substantive differences in latent profile structure between male and female patients. Accordingly, the present study neither forces the determination of a single optimal profile model within the sex-stratified LPA nor interprets this null result as evidence that the latent profile structures of the two sexes are equivalent. The principal value of this exploratory analysis lies in delineating the feasibility boundaries of sex-stratified LPA and providing preliminary methodological reference for future larger-scale sex-comparative investigations.

### 3.5 Univariate analysis of factors associated with optimal latent profiles in the full sample

Using latent profile membership (low-level group vs. high-level group) as the grouping variable, univariate analyses were conducted for each sociodemographic characteristic, clinical feature, and core psychosocial variable. Chi-square tests were employed for categorical variables, and independent-samples t-tests were used for continuous variables. The results of the univariate analyses are presented in detail in Table 6.

**Table 6:** Univariate analysis of differences across latent profile classes.

Variables	Items	Profile class 1	Profile class 2	χ^2^/t	*p*
Gender	Male	156	184	1.184	0.277
	Female	60	56		
Marital status	Married	137	174	4.356	0.226
	Unmarried	25	20		
	Divorced	30	26		
	Widowed	24	20		
Educational background	Primary school and below	54	85	7.711	0.021
	Junior high school - High school	88	96		
	Bachelor’s degree and above	74	59		
Employment status	Retirement	49	60	1.269	0.530
	Unemployed	73	88		
	Employed	94	92		
Place of residence	Urban	155	136	11.215	<0.001
	Rural	61	104		
Monthly personal income	≤4,000 RMB	109	91	7.409	0.025
	4,001–8,000 RMB	65	94		
	≥ 8,001 RMB	42	55		
Liver cancer stage	Stage I	33	27	8.375	0.039
	Stage II	71	58		
	Stage III	76	113		
	Stage IV	36	42		
Child–Pugh classification of liver function	A classification	105	116	0.692	0.707
	B classification	76	91		
	C classification	35	33		
Cirrhosis status	Yes.	141	150	0.38	0.538
	No.	75	90		
Time since diagnosis	≤6 month	81	63	6.791	0.034
	6–12 month	50	62		
	≥1 years	85	115		
Line of antitumor therapy	Not receiving treatment	17	27	4.029	0.258
	One line	89	80		
	Two line	62	80		
	Three line and above	48	53		
Smoking behavior	Yes.	131	120	5.209	0.022
	No.	85	120		
Alcohol behavior	Yes.	110	94	6.359	0.012
	No.	106	146		
Age	M ± SD	56.25 ± 8.691	57.40 ± 8.704	−1.405	0.161
Cancer-related fatigue	M ± SD	3.674 ± 0.497	3.366 ± 0.531	6.370	<0.001
Social support	M ± SD	3.067 ± 0.554	3.457 ± 0.565	−7.428	<0.001

Regarding sociodemographic characteristics, statistically significant distributional differences between the two latent profiles were observed for educational background (χ^2^ = 7.711, *p* = 0.021), place of residence (χ^2^ = 11.215, *p* < 0.001), and monthly personal income (χ^2^ = 7.409, *p* = 0.025). In contrast, Gender (χ^2^ = 1.184, *p* = 0.277), marital status (χ^2^ = 4.356, *p* = 0.226), employment status (χ^2^ = 1.269, *p* = 0.530), and age (t = −1.405, *p* = 0.161) did not differ significantly between the two profiles, suggesting that these variables did not exert a significant discriminatory role in determining latent profile membership for food and nutrition literacy and diet self-efficacy among liver cancer patients in the present sample.

With respect to clinical characteristics, statistically significant distributional differences between the two latent profiles were identified for liver cancer stage (χ^2^ = 8.375, *p* = 0.039), time since diagnosis (χ^2^ = 6.791, *p* = 0.034), smoking behavior (χ^2^ = 5.209, *p* = 0.022), and alcohol consumption (χ^2^ = 6.359, *p* = 0.012). However, Child–Pugh classification of liver function (χ^2^ = 0.692, *p* = 0.707), cirrhosis status (χ^2^ = 0.380, *p* = 0.538), and line of antitumor therapy (χ^2^ = 4.029, *p* = 0.258) did not differ significantly between the two profiles.

Regarding core psychosocial variables, both cancer-related fatigue and social support exhibited highly significant between-group differences across the two latent profiles. The mean item score for cancer-related fatigue in the low-level group (M = 3.674, SD = 0.497) was significantly higher than that in the high-level group (M = 3.366, SD = 0.531; t = 6.370, *p* < 0.001), whereas the mean item score for social support in the low-level group (M = 3.067, SD = 0.554) was significantly lower than that in the high-level group (M = 3.457, SD = 0.565; t = −7.428, *p* < 0.001).

### 3.6 Binary logistic regression analysis of latent profile membership

To further delineate the independent predictive effects of each factor on latent profile membership and to evaluate the net effects of key factors while controlling for other potential confounders, binary logistic regression was performed using the two-profile classification derived from the full sample as the dependent variable, with the “high food and nutrition literacy–high diet self-efficacy” group designated as the reference category. All variables that achieved statistical significance in the univariate analyses were simultaneously entered into the binary logistic regression model. The regression results are presented in Table 7.

**Table 7:** Binary logistic regression analysis of food and nutrition literacy and diet self-efficacy latent profile membership.

Variables	Items	B	SE	Wald χ^2^	*p*	OR	LLCI	ULCI
Cancer-related fatigue		0.841	0.228	13.552	<0.001	2.319	1.482	3.628
Social support		−1.078	0.205	27.716	<0.001	0.34	0.228	0.508
Educational background	Primary school and below	0.414	0.259	2.548	0.11	1.513	0.91	2.516
	Junior high school - High school	0.705	0.284	6.149	0.013	2.023	1.159	3.53
	Bachelor’s degree and above (refer)							
Place of residence	Urban	0.725	0.231	9.862	0.002	2.065	1.313	3.248
	Rural (refer)							
Monthly personal income	≤4,000 RMB	0.575	0.29	3.921	0.048	1.777	1.006	3.139
	4,001–8,000 RMB	0.014	0.302	0.002	0.963	1.014	0.561	1.834
	≥ 8,001 RMB (refer)							
Liver cancer stage	Stage I	0.188	0.393	0.229	0.632	1.207	0.559	2.607
	Stage II	0.506	0.331	2.336	0.126	1.659	0.867	3.176
	Stage III	−0.088	0.308	0.082	0.774	0.915	0.5	1.675
	Stage IV (refer)							
Time since diagnosis	≤6 month	0.334	0.287	1.357	0.244	1.397	0.796	2.451
	6–12 month	−0.327	0.271	1.459	0.227	0.721	0.424	1.226
	≥1 years (refer)							
Smoking behavior	Yes.	−0.374	0.219	2.917	0.088	0.688	0.448	1.057
	No. (refer)							
Alcohol behavior	Yes.	−0.345	0.22	2.473	0.116	0.708	0.46	1.089
	No. (refer)							

Among the core psychosocial variables, cancer-related fatigue exerted a significant independent positive predictive effect on latent profile membership (B = 0.841, *p* < 0.001, OR = 2.319, 95% CI = [1.482, 3.628]). This finding indicates that, after controlling for other covariates, each one-unit increase in cancer-related fatigue was associated with an approximately 131.9% increase in the odds of being classified into the “low-level group” relative to the “high-level group.” Social support exerted a significant independent negative predictive effect on latent profile membership (B = −1.078, *p* < 0.001, OR = 0.340, 95% CI = [0.228, 0.508]), indicating that each one-unit increase in perceived social support was associated with an approximately 66.0% reduction in the odds of being classified into the “low-level group.” Thus, cancer-related fatigue serves as a risk factor that significantly increases the likelihood of membership in the low-level profile, whereas social support functions as a protective factor that significantly attenuates this risk.

The effect of educational attainment exhibited a partially significant pattern. Using a bachelor’s degree or above as the reference category, patients with a junior high school to senior high school education had significantly higher odds of being classified into the “low-level group” (B = 0.705, *p* = 0.013, OR = 2.023, 95% CI = [1.159, 3.530]), indicating that the risk of membership in the low-level profile for patients with a junior-to-senior high school education was approximately 2.023 times that of patients with a bachelor’s degree or above. However, the difference between patients with a primary school education or below and those with a bachelor’s degree or above did not reach statistical significance (*p* = 0.110). The effect of place of residence was statistically significant (B = 0.725, *p* = 0.002, OR = 2.065, 95% CI = [1.313, 3.248]); using rural residence as the reference category, liver cancer patients residing in urban areas had approximately 2.065 times the odds of being classified into the “low-level group” compared with their rural counterparts. Regarding monthly personal income, using an income above 8,000 RMB as the reference category, patients with a monthly income of 4,000 RMB or below had significantly elevated odds of being classified into the “low-level group” (B = 0.575, *p* = 0.048, OR = 1.777, 95% CI = [1.006, 3.139]), whereas the difference between the 4,001–8,000 RMB income group and the reference group was not statistically significant (*p* > 0.05), suggesting that the influence of economic resources on profile membership may be primarily manifested between the two extremes of the income spectrum. The differences between each liver cancer stage category and the reference category (stage IV) did not reach statistical significance (all *p* > 0.05), nor did the differences between each time-since-diagnosis category and the reference category (≥ 1 year; all *p* > 0.05). Furthermore, the independent predictive effects of smoking behavior (*p* > 0.05) and alcohol consumption behavior (*p* > 0.05) on profile membership failed to attain statistical significance under multivariable control.

## 4 Discussion

### 4.1 Latent profile analysis of food and nutrition literacy and diet self-efficacy

The present study identified two latent profile types among the full sample of liver cancer patients. These findings indicate that food and nutrition literacy and diet self-efficacy among liver cancer patients tend to coalesce into co-directionally coupled competency–belief configurations—that is, individuals with stronger nutritional information processing capacities also tend to possess greater confidence in executing dietary behaviors, and vice versa. According to social cognitive theory, higher nutritional cognitive capacity facilitates the construction of clear behavioral outcome expectations, which in turn enhances patients’ confidence in executing behaviors when confronted with complex dietary barrier situations; stronger self-efficacy further motivates patients to proactively seek out and deeply internalize nutritional knowledge. Conversely, patients with a weak nutritional cognitive foundation, when facing complex dietary barriers such as treatment-related appetite loss, nausea, and metabolic disturbances, tend to exhibit pronounced behavioral efficacy deficits, giving rise to a vicious cycle in which nutritional cognitive deficiency and diminished dietary behavioral motivation mutually reinforce one another, thereby making intermediate states difficult to sustain in dynamic equilibrium.

It is noteworthy that the present study did not identify cross-type profiles such as “high nutrition literacy–low self-efficacy” or “low nutrition literacy–high self-efficacy.” This may be attributable to the fact that, under the symptom burden of treatment-related adverse effects, fatigue, and appetite decline, liver cancer patients who lack sufficient nutrition literacy often find it difficult to formulate concrete and actionable dietary strategies, rendering self-efficacy difficult to maintain. Nonetheless, future research may consider employing more refined measurement indicators—such as separating nutritional knowledge, label-reading skills, planning abilities, and objective indices of actual dietary behavior—or incorporating longitudinal data to examine whether cross-type profiles emerge at different time points or treatment stages.

### 4.2 Gender-stratified latent profile comparative analysis

The present study independently conducted latent profile analyses within male and female liver cancer patient subsamples to systematically examine subgroup heterogeneity in the joint dimensions of food and nutrition literacy and diet self-efficacy under a sex-stratified analytical framework. The results revealed a distinctive pattern of considerable methodological discussion value: across both male and female samples, the LMR-LRT failed to reach statistical significance in all pairwise comparisons of adjacent models, rendering the conventional strategy of using the LMR-LRT to identify the optimal number of profiles unable to provide clear and consistent statistical support, and making it difficult to determine the optimal number of profiles based on any single statistical criterion. This may be attributable to the substantial reduction in subsample sizes following sex stratification relative to the full sample, which imposed meaningful constraints on the statistical power of the LMR-LRT under small-sample conditions, limiting its capacity to reliably discriminate the significance of differences between adjacent profile models. Additionally, the persistent significance of the BLRT essentially reflects the statistical superiority of more complex models over more parsimonious models at the log-likelihood level; however, under conditions of relatively limited sample sizes, this test exhibits heightened sensitivity to increases in model complexity, readily producing a pattern of persistent significance as the number of profiles increases, which does not necessarily imply that additional profiles possess substantive explanatory value and should not be used as the sole basis for determining the optimal number of profiles. Furthermore, entropy values for all candidate models remained at high levels in both sex-stratified samples, indicating that models with different numbers of profiles all achieved relatively clear individual classification, which further undermined the operational utility of entropy differences as a decisive discriminating criterion.

### 4.3 Analysis of influencing factors

Cancer-related fatigue leads to cognitive resource depletion and behavioral maintenance failure. Cancer-related fatigue encompasses not merely physical decline but also cognitive fatigue and emotional fatigue components. Its impact on food and nutrition literacy may operate through two mechanisms. First, fatigue impairs sustained attention and executive function, making it difficult for patients to continuously retrieve, comprehend, and evaluate nutritional information, with particularly pronounced effects on tasks requiring multi-step processing. Second, fatigue diminishes patients’ initiative in information acquisition and learning, reducing the frequency of interactions with dietitians, proactive questioning, and self-monitoring, thereby establishing a negative cycle from low learning engagement to low mastery. Consequently, when patients repeatedly experience dietary planning failures under the compounding influence of treatment-related nausea, appetite loss, abdominal distension, and fatigue, their self-efficacy is susceptible to frustration-driven downward revision, ultimately evolving into avoidant or abandonment-oriented coping.

#### 4.3.1 Social support

According to the stress-buffering model of social support, adequate social support can indirectly preserve patients’ cognitive functional reserves by buffering the intensity of disease-related stress responses and attenuating perceived fatigue severity, thereby establishing a favorable cognitive environmental foundation for maintaining higher levels of food and nutrition literacy. Concurrently, emotional encouragement and positive feedback from family members and the healthcare team can strengthen patients’ beliefs and intrinsic motivation to overcome dietary barriers through vicarious experience and verbal persuasion mechanisms, thereby enhancing diet self-efficacy. These findings deepen and extend, to a certain extent, the evidence base regarding the mechanisms linking social support to nutritional self-management behaviors among cancer patients, and further substantiate the necessity and feasibility of systematically integrating social support interventions into nutritional management programs for liver cancer patients.

#### 4.3.2 Educational background

Patients’ educational attainment exhibited a partially significant nonlinear effect pattern on latent profile membership. Patients with a bachelor’s degree or above, by virtue of their stronger information processing abilities, critical thinking literacy, and systematic health management awareness, demonstrated clear cognitive advantages in proactively acquiring, critically comprehending, and flexibly applying complex nutritional information—a finding consistent with research conclusions in the health literacy field regarding the positive association between higher educational attainment and greater health-related information literacy. However, the difference between patients with a primary school education or below and those with a bachelor’s degree or above did not reach statistical significance. This may be because patients with lower educational attainment have accumulated relatively rich traditional food and dietary cultural knowledge through long-term life experience and rely to a greater extent on instrumental and informational support provided by family members to compensate for the nutritional cognitive limitations arising from insufficient formal education. By contrast, the junior-to-senior high school education group may occupy a cognitive “middle layer” characterized by relatively limited knowledge accumulation and insufficient autonomous information-seeking capacity, coupled with a lower propensity to proactively seek family-based supplementary support, resulting in a relative intermediate disadvantage in nutritional behavioral competency.

#### 4.3.3 Place of residence

The present study found that the risk of membership in the low-level profile was significantly higher among urban-dwelling patients than among rural-dwelling patients. This may be attributable to the fact that urbanized lifestyles—characterized by higher frequencies of dining out, greater reliance on ultra-processed foods, and the compression of dietary planning time by fast-paced living—may create more formidable practical barriers for urban patients attempting to adhere to healthy dietary regimens during treatment, thereby increasing the situational complexity of challenges to diet self-efficacy. This finding suggests the adoption of more nuanced multidimensional urbanization measurement instruments, combined with dietary environment assessments, treatment intensity indicators, and fatigue burden measures, to more precisely elucidate the differentiated pathways through which place of residence influences nutritional behavioral capacity among liver cancer patients.

#### 4.3.4 Personal monthly income

Patients with a monthly income of 4,000 RMB or below had a significantly higher risk of membership in the low-level profile compared with those earning above 8,000 RMB, whereas the difference between the 4,001–8,000 RMB income group and the reference group was not statistically significant. This finding suggests that lower economic resources may systematically constrain patients’ nutritional behavioral capacity. Constrained by limited purchasing power, low-income patients face greater practical barriers to accessing diverse, high-quality healthy food ingredients. Economic stress itself may indirectly deplete the cognitive and emotional resources necessary for engagement in healthy dietary management by elevating patients’ chronic psychological burden and daily stress levels. Simultaneously, low-income patients typically have relatively limited access to systematic nutrition education and professional dietary counseling services, further constraining the possibilities for nutritional knowledge accumulation and dietary skill development. This finding resonates with theoretical frameworks in the health inequality field regarding the multidimensional pathways through which socioeconomic status influences health-related behavioral capacities, and highlights the importance of fully considering equity in economic accessibility when designing nutritional intervention programs for liver cancer patients.

It is noteworthy that liver cancer stage, time since diagnosis, smoking behavior, and alcohol consumption behavior all exhibited significant between-group differences in the univariate analyses but failed to maintain statistical significance after controlling for other covariates in the binary logistic regression model. This finding suggests that the apparent effects of these clinical and behavioral variables on profile membership are largely explainable by more proximal variables such as cancer-related fatigue, social support, and socioeconomic factors. For example, advanced liver cancer staging is often accompanied by more severe cancer-related fatigue, whereas longer time since diagnosis may be associated with the accumulation of richer social support resources; the effects of smoking and alcohol consumption may be absorbed through their covariation with educational attainment, socioeconomic status, and overall health consciousness. This finding statistically underscores the central role of cancer-related fatigue and social support as proximal psychosocial variables in predicting nutritional behavioral profile membership among liver cancer patients, while also suggesting that clinical intervention resources should be preferentially allocated to the two key domains of fatigue management and social support enhancement.

### 4.4 Implications for clinical practice, malnutrition prevention, and health policy

Given that nearly half of liver cancer patients simultaneously exhibited dual low-level status in both food and nutrition literacy and diet self-efficacy, clinical dietitians and oncology nursing teams conducting nutritional assessments should attend not only to objective nutritional status indicators but should also incorporate food and nutrition literacy and diet self-efficacy into routine nutritional screening and assessment systems. After identifying high-risk patients belonging to the “low–low” profile through brief screening instruments, integrated nutritional intervention programs emphasizing both knowledge empowerment and efficacy enhancement can be developed: on the one hand, structured nutrition education to improve food and nutrition literacy, and on the other hand, psychobehavioral strategies such as motivational interviewing, progressive goal setting, and peer support groups to enhance perceived diet self-efficacy—rather than treating the two as separate, independent intervention modules.

The identification of cancer-related fatigue as the strongest risk factor carries direct clinical value as an intervention target. Given the robust negative predictive effect of fatigue on food and nutrition literacy and diet self-efficacy, patients’ fatigue status should be fully considered in the design and implementation of nutritional intervention programs. For patients experiencing severe fatigue, traditional concentrated nutrition education sessions may yield suboptimal results due to cognitive resource depletion; segmented, modular educational formats may instead be considered, utilizing time windows when patients’ energy levels are relatively replenished for targeted education. Concurrently, integrating fatigue management strategies can mitigate fatigue’s inhibitory effects on nutritional behavioral capacity at the source. Furthermore, clinical teams evaluating nutritional intervention outcomes should monitor fatigue levels as an important effect-moderating variable, avoiding the simplistic attribution of low intervention responsiveness among severely fatigued patients to poor adherence.

The identification of social support as the most important protective factor provides a rationale for integrating social relationship networks into intervention strategies. In clinical practice, patients’ primary caregivers should be encouraged to participate in the full course of nutrition education and skill training, enabling them to serve as informed assistants and emotional supporters in patients’ daily dietary management. Additionally, establishing peer support networks centered on patients with the same cancer type can effectively enhance patients’ diet self-efficacy through the demonstration effects of successful management cases and emotional resonance among peers. Healthcare institutions may also leverage mobile health platforms or online communities to provide continuous remote nutritional support and social interaction functionality for liver cancer patients after discharge, compensating for the potential attenuation of social support in the post-discharge setting.

For liver cancer patients with lower educational attainment, urban residence with higher treatment intensity, and lower personal monthly income, clinical nutritional intervention programs should be fully adapted to their cognitive characteristics, cultural backgrounds, and economic conditions, with differentiated precision intervention strategies implemented accordingly. Specific measures include the adoption of illustrated, accessible, and tiered nutritional education materials to lower the cognitive processing threshold for nutritional information; the provision of specific, actionable, low-cost, and nutrient-dense recipes and food substitution plans; the comprehensive integration of extended service functions from primary healthcare institutions and internet-based healthcare platforms to reduce the equity impact of geographic accessibility disparities on nutritional service access; and the active advocacy for incorporating professional nutritional management services into oncology patient medical assistance programs and basic medical insurance systems, thereby safeguarding equitable access to professional nutritional support services for low-income liver cancer patients at the institutional and policy levels.

The finding that sex-stratified analysis did not yield an optimal profile solution also carries practical reference value. This finding suggests, to a certain extent, that under current sample conditions, the core framework of nutritional intervention programs for liver cancer patients may not require fundamental structural modifications on the basis of sex alone. When designing nutritional interventions, clinicians may direct greater attention toward cancer-related fatigue level, social support status, educational attainment, and economic conditions, stratification variables with stronger predictive power, using these factors rather than sex alone as the primary basis for individualized intervention adjustments. This does not, of course, imply the complete disregard of sex sensitivity, but rather recommends that when prioritizing interventions in resource-constrained clinical environments, the aforementioned core influencing factors be accorded higher priority.

Importantly, the present findings should be interpreted in relation to the prevention and management of malnutrition in oncology care. Patients with liver cancer are highly susceptible to malnutrition due to impaired hepatic metabolism, treatment-related symptoms, appetite loss, systemic inflammation, and reduced dietary intake. The “low food and nutrition literacy–low diet self-efficacy” profile identified in this study may therefore represent a behavioral vulnerability phenotype for nutrition-related deterioration. Patients in this group may have difficulty understanding dietary recommendations, selecting nutrient-dense foods, managing nutrition-impact symptoms, and maintaining adequate intake under fatigue or psychosocial stress. Thus, food and nutrition literacy and diet self-efficacy should be incorporated into routine nutritional risk assessment together with conventional clinical indicators such as weight loss, dietary intake, body mass index, muscle loss, and laboratory markers where appropriate. Early identification of this vulnerable subgroup may allow oncology nurses, dietitians, and physicians to provide timely, individualized, and multidisciplinary interventions before severe malnutrition develops.

At the policy level, these findings highlight the need to strengthen standardized oncology nutrition services. Hospitals and health systems should establish continuous nutrition screening, referral, counseling, and follow-up pathways for patients with liver cancer across hospitalization, outpatient treatment, discharge, and community-based survivorship care. Given the observed effects of income, social support, and fatigue, policy makers should also consider improving reimbursement for nutrition counseling and medical nutrition therapy, developing caregiver-inclusive education programs, expanding community-based nutrition support, and providing targeted assistance for economically disadvantaged patients. Such equity-oriented policies may help reduce malnutrition risk and improve quality of life among patients with liver cancer.

### 4.5 Limitations and future research directions

Despite the aforementioned findings, the present study has several notable limitations and deficiencies.

First, the present study employed a cross-sectional survey design, and the data obtained reflect only static cross-sectional information at a single time point, without any intervention component or follow-up assessment, precluding causal inference or the determination of temporal effects among variables. Although cancer-related fatigue was identified as an independent risk factor for membership in the low food and nutrition literacy–low diet self-efficacy profile, the temporal direction of this association cannot be determined. Fatigue may reduce patients’ attention, motivation, and ability to acquire and apply nutrition-related information, thereby weakening food and nutrition literacy and diet self-efficacy. Future research should adopt prospective longitudinal tracking designs to assess changes in nutritional cognition and behavioral capacity among liver cancer patients at multiple time points and employ latent transition analysis to examine dynamic transition patterns between profiles and their predictive factors.

Second, the present study recruited participants via convenience sampling from three tertiary-level hospitals, and the representativeness of the sample is inherently limited. Patients at tertiary hospitals may differ systematically from liver cancer patients managed at primary-level hospitals or in community settings with respect to disease severity, healthcare resource accessibility, and exposure to health education opportunities. Therefore, the identified two-profile solution and its associated factors should be generalized to other regions, healthcare levels, and cultural contexts with caution. Future studies should consider adopting multi-center, multi-level sampling strategies that incorporate liver cancer patient samples from healthcare institutions of varying levels and from diverse geographic regions to enhance the external validity of the findings.

Third, the subsample sizes within each sex-stratified subgroup were relatively limited, which may constitute an important technical reason for the inability of the sex-stratified latent profile analyses to identify an optimal solution. Limited sample sizes not only reduce the precision of model parameter estimates and the sensitivity of statistical tests but also constrain the effective testing of models specifying larger numbers of profiles. Future research should ensure adequate sample sizes before conducting sex-stratified analyses.

Fourth, the core variables in the present study were all measured through patient self-report questionnaire instruments. Although Harman’s single-factor test indicated that common method bias did not pose a serious threat, the inherent social desirability bias, recall bias, and subjectivity associated with self-report measurement may nonetheless affect data accuracy to some extent. Such bias may lead to overestimation or underestimation of the associations among food and nutrition literacy, diet self-efficacy, fatigue, and social support. Future research may consider incorporating objective measurement indices in a complementary multi-method assessment system with self-report instruments to enhance comprehensive measurement validity.

Fifth, the present study focused on food and nutrition literacy and diet self-efficacy as latent profile indicators, but did not include objective nutritional outcomes as profile indicators or distal outcomes. Therefore, although the low food and nutrition literacy–low diet self-efficacy profile may represent a behavioral vulnerability phenotype relevant to malnutrition risk, this study cannot directly determine whether this profile predicts actual malnutrition, sarcopenia, cachexia, treatment interruption, postoperative complications, hospitalization duration, quality of life, or survival outcomes. Future research should examine the predictive validity of these latent profiles by linking profile membership to clinically meaningful nutritional and oncological outcomes. Such evidence would strengthen the clinical utility of profile-based risk stratification.

## 5 Conclusion

The present study was conducted with 456 hospitalized and outpatient liver cancer patients. The full-sample LPA results supported the two-profile model as the optimal solution, identifying two latent subgroup types with internal coherence: the low food and nutrition literacy–low diet self-efficacy type (47.37%) and the high food and nutrition literacy–high diet self-efficacy type (52.63%). At the level of predictive factors for profile membership, cancer-related fatigue was confirmed as an independent risk factor for classification into the low-level profile, and social support was confirmed as an independent protective factor. Lower educational attainment, urban residence, and lower personal monthly income also increased the risk of low-level profile membership to varying degrees. At the exploratory supplementary analysis level, analyses within both sex-stratified subgroups exhibited a consistent statistical pattern of continuously declining information criteria, persistently significant BLRT, and consistently non-significant LMR-LRT, precluding unambiguous confirmation of the optimal number of profiles via commonly employed statistical standards. In sum, the present study made clinically meaningful progress in identifying the latent subgroup type structure of food and nutrition literacy and diet self-efficacy among liver cancer patients and in clarifying the key driving factors, providing a preliminary evidence-based framework for subgroup-targeted intervention research in the field of oncology nutritional management.

## Data Availability

The raw data supporting the conclusions of this article will be made available by the authors, without undue reservation.
